# Thermoreversible Cross-Linked Rubber Prepared via Melt Blending and Its Nanocomposites

**DOI:** 10.1021/acsapm.2c00416

**Published:** 2022-06-17

**Authors:** Francesco Cantamessa, Giacomo Damonte, Orietta Monticelli, Rossella Arrigo, Alberto Fina

**Affiliations:** † Dipartimento di Scienza Applicata e Tecnologia, Politecnico di Torino, 15121 Alessandria, Italy; ‡ Dipartimento di Chimica e Chimica Industriale, Università di Genova, 16146 Genova, Italy

**Keywords:** recyclable rubber, Diels−Alder cross-linked polymer, vitrimer, covalent adaptable network, thermoreversible cross-linking, thermally conductive rubber

## Abstract

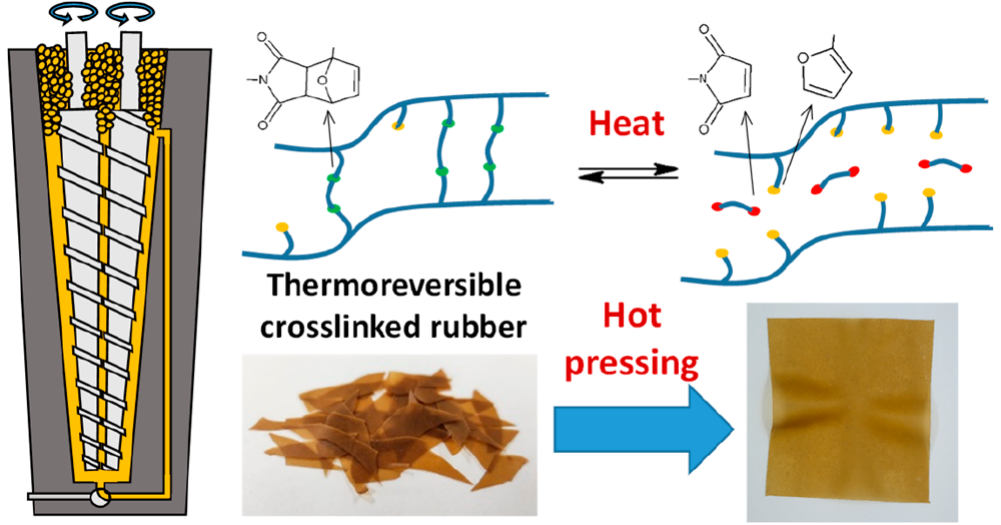

A covalent adaptable network based on the thermoreversible cross-linking of an ethylene–propylene rubber through Diels–Alder (DA) reaction was prepared for the first time through melt blending as an environmental-friendly alternative to traditional synthesis in organic solvents. Functionalization of the rubber with furan groups was performed in a melt blender and subsequently mixed with different amounts of bismaleimide in a microextruder. Cross-linking was confirmed by FT-IR spectroscopy and insolubility at room temperature, while its thermoreversible character was confirmed by a solubility test at 110 °C and by remolding via hot-pressing. Mechanical and thermomechanical properties of the obtained rubbers showed potential to compete with conventionally cross-linked elastomers, with stiffness in the range 1–1.7 MPa and strain at break in the range 200–500%, while allowing recycling via a simple melt processing step. Nanocomposites based on the thermoreversible rubber were prepared with reduced graphene oxide (rGO), showing significantly increasing stiffness up to ca. 8 MPa, ∼2-fold increased strength, and thermal conductivity up to ∼0.5 W/(m K). Results in this paper may open for industrially viable and sustainable applications of thermoreversible elastomers.

## 1 Introduction

Covalent cross-linking technology in rubber has been well-established for decades. Cross-linked rubbers present higher toughness, deformability, and thermal stability than their thermoplastic homologues. In addition, a cross-linked network cannot be dissolved in solvents, thus providing chemical resistance even in harsh conditions. However, the increasing attention for materials’ sustainability is driving the development of cross-linked yet recyclable materials via reversibly cross-linked networks.

Reversible cross-linking can be achieved through a wide range of reactions:^1^ transesterification,^2−4^ transamination,^5,6^ dioxaborolane^7,8^ disulfide metathesis,^9^ and disulfide–thiol exchange,^10^ among others. In this scenario, significant research attention focused on thermally reversible networks, which present covalent cross-links among polymer chains that are cleavable at high temperature, opening the way for reprocessing of cross-linked networks, similarly to thermoplastic polymers.

Thermally reversible Diels–Alder (DA) networks are one of the possible options.^11−19^ In DA cycloaddition, one compound with two conjugated double bonds, the diene, and another compound with a weak double bond, the dienophile, react to form a cyclic adduct. The DA reaction is favored up to 60 °C, while the cleavage of the DA adduct and the new formation of diene and dienophile (retro-DA reaction) occur at about 110 °C.^20^ This reaction has been exploited to prepare covalent adaptable networks with several polymers, including polystyrene,^11^ polyketones,^14^ and polyurethane,^19,21^ with the main aim of obtaining reprocessable and self-healable networks.

In the field of rubbers, the Diels–Alder reaction has been used to prepare an acrylic dielectric elastomer with thermotunable stiffness^22^ for application in soft functional materials.^22,23^ Natural rubber^24^ and polybutadiene rubber^25^ have also been thermoreversibly cross-linked via DA reaction through a solvent process, bringing interesting perspectives for tire recycling. With the aim of forming a thermoreversible elastomer, Polgar et al. reported the functionalization of ethylene–propylene–diene^16,18^ and ethylene–vinyl acetate^17^ rubbers with a furan group, in the role of diene, reacting furfurylamine (FFA) with anhydride groups grafted on the chain. The functionalized polymer was then cross-linked by the DA reaction using a bismaleimide (BM), in the role of a bifunctional dienophile, leading to a reversibly cross-linked rubber with excellent mechanical strength, insoluble at room temperature but cleavable once the temperature sufficient to activate retro-DA was reached.

In addition to cross-linking, rubbers are usually reinforced with fillers to further enhance their stiffness and strength.^26^ Traditionally, the most commonly used fillers are carbon black and silica, but other nanofillers such as graphene and related materials^27^ were also proposed, taking advantage of their high specific surface area. With regard to thermoreversible networks, nanofillers prefunctionalized with diene or dienophile (graphene oxide^28−30^ and silica^31^) have been used to prepare nanocomposites, involving these fillers in the cross-linking process. It has also been reported in the literature that the DA reaction can take place on sp^2^-hybridized carbon particles, such as carbon nanotubes^32^ or graphene.^33^ This possibility has been explored in nanocomposites where fillers were used as a source of diene^34,35^ or dienophile^36,37^ sites, without the need to prefunctionalize these fillers.

Building on the described state of the art, this work addressed for the first time the preparation of a thermoreversibly cross-linked ethylene–propylene-based rubber via melt processing. Indeed, both the grafting of furan on the main chain and the cross-linking with BM were performed without the use of solvents by reactive melt blending. Furthermore, the properties of the thermoreversibly cross-linked rubber were further modified by the dispersion of reduced graphene oxide (rGO) to enhance stiffness, strength, and thermal conductivity of the rubber. The development and validation of melt processing methods reported in this work guarantee environmentally friendly and cost-effective production of mechanically reinforced and thermoreversible elastomers, paving the way for sustainable and industrially viable applications.

## 2 Materials and Methods

### 2.1 Materials

Ethylene–propylene rubber grafted with maleic anhydride (EPRgMA) is a commercial product (Keltan 1519R) with about 2 wt % of grafted maleic anhydride (MA), kindly provided by ARLANXEO Performance Elastomers (Netherlands). Irganox 1010 was purchased by BASF (Germany). Furfurylamine (FFA, ≥99%), acetone (≥99.8%), and toluene (≥99%) were purchased by Sigma-Aldrich and used without any distillation step. 2,2-Bis[4-(4-maleimidophenoxy)phenyl]propane (BM) was purchased by TCI Chemicals and used as received. The thermally reduced graphene oxide (rGO) was provided by Avanzare Innovacion Tecnologica S.L. (Spain). The synthetic procedure was previously reported;^38^ in brief, rGO was prepared by oxidation of natural graphite, tip sonication in water solution, and then thermal reduction at 1060 °C in an argon atmosphere. rGO nanoflakes are tens of micrometers wide and a few nanometers thick. Full characterization has been described elsewhere.^39^

### 2.2 Melt Processing of Polymers and Polymer Nanocomposites

#### 2.2.1 Preparation of Furan-Grafted EPR

Grafting of furan groups on the rubber chains was performed through the well-known reaction between anhydride and amine groups.^40^ In the field of covalent adaptable networks, the reaction between anhydrides and furfurylamine was previously reported^16,41^ in solvent media. In this work, EPRgMA was grafted with furan groups through reaction with FFA in a W50E internal mixer (Brabender, Germany). The mixer was set at 150 °C and filled with EPRgMA at 30 rpm screw rotation speed, together with an antioxidant (Irganox 1010), in the amount of 0.1 wt % of EPRgMA. After that, FFA was added in a 2:1 molar ratio with MA groups in EPRgMA, directly into the molten rubber and mixed for 5 min at 60 rpm. The excess amine was designed to compensate for its partial volatilization during melt blending. The obtained compound, termed EPRgF*, was manually chopped and annealed at 180 °C and 75 mbar in a vacuum oven for 15 min to complete the reaction and remove the excess FFA. The compound obtained after annealing is termed EPRgF. A sample of EPRgMA was subjected to the same annealing treatment in a vacuum oven to verify the condensation of the hydrolyzed dicarboxylic acid form, which may be obtained even in ambient conditions,^42^ to anhydride. The obtained product is termed EPRgMA_ann.

#### 2.2.2 Thermoreversible Cross-Linking of EPRgF

EPRgF was blended with BM in different ratios in a corotating twin screw microextruder (DSM Xplore 15, Netherlands) for 5 min at 150 °C and 100 rpm. Table 1 reports the compositions of the blends developed for different molar ratios between furan and maleimide functions, assuming all the anhydride sites of EPRgMA were previously reacted with FFA. After blending, the compound was extruded from the chamber and collected. Attempts to further increase the BM content resulted in extruder blockage because of the high viscosity.

**Table 1:** Compositions of EPRgF-BM Blends and EPRgF-BM-rGO Nanocomposites

formulation code	furan:maleimide molar ratio	BM content (wt %)
EPRgF-2.7%BM	1:0.5	2.7
EPRgF-5.4%BM	1:1	5.4
EPRgF-8.1%BM	1:1.5	8.1

formulation code	EPRgF (wt %)	BM content (wt %)	rGO content (wt %)
EPRgF-5.4%BM/5%rGO	89.9	5.1	5.0
EPRgF-5.4%BM/10%rGO	85.1	4.9	10.0

For characterization, the different compounds were hot-pressed at 150 °C for 4 min to produce films of the required shape and thickness. To ensure completion of cross-linking, all the materials were subjected to annealing in an oven for 3 days at 50 °C, according to a previously reported procedure.^16^

#### 2.2.3 Nanocomposite Preparation

EPRgF-5.4%BM was selected to prepare rGO nanocomposites, with compositions reported in Table 1. The necessary amount of rGO (0.6 or 1.2 g) was ultrasonicated in 150 mL of acetone via tip sonication at 150 W and 20 kHz (Sonics, Vibra-cel-VCX-500, 13 mm tip, USA) for 30 min, through steps of 5 s ON and 5 s OFF, with a beaker immersed in an water/ice bath to avoid overheating. Then, the requested amount of BM was dissolved in the suspension, and the solvent was evaporated at room temperature in fume hood overnight, under magnetic stirring. The obtained mixtures of rGO and BM were finally melt blended with EPRgF with the same conditions used for EPRgF-BM products, namely 5 min of mixing at 150 °C at 100 rpm, and then extruded. In the case of 10% rGO, where the high viscosity led to blockage of the extruder after ∼2 min, extruding was not possible, requiring manual recovery of the composite from the mixing chamber. The nanocomposites were hot-pressed at 150 °C for 4 min to produce specimens for characterization and annealed in an oven at 50 °C for 3 days.

### 2.3 Characterization Methods

Differential scanning calorimetry (DSC) tests were performed with a Q20 DSC (TA Instruments, USA) on samples of ca. 8 mg in closed aluminum pans with perforated lids. The measurements were performed under dry N_2_ gas, running a heating ramp from 0 to 200 °C, then a cooling ramp to 0 °C, and another heating ramp up to 200 °C. Both heating and cooling ramp were performed at a 10 °C/min rate.

Attenuated total reflection Fourier transformed infrared (ATR-FTIR) spectroscopy was performed by using a Frontier spectrometer (PerkinElmer, USA), running 16 scans with a resolution of 4 cm^–1^, on the surface of chopped samples.

^1^H NMR proton spectroscopy was performed by using a Varian “Mercury 300” at a frequency of 300 MHz. Samples previously dissolved in CDCl_3_ at a concentration of 30 mg/mL were analyzed in 10 mm NMR tubes at room temperature.

The morphology of the samples was studied by scanning electron microscopy (SEM) using an EVO 15 SEM (Zeiss, Germany) with a beam voltage of 20 kV. The micrographs were taken from the fragile fracture surfaces obtained after soaking the samples in liquid nitrogen for 2 min.

Dynamic mechanical thermal analysis (DMTA) measurements were performed by using a Q800 DMTA (TA Instruments, USA) equipped with tension film clamps. The 8 × 16 mm^2^ specimens were cut from hot-pressed 1 mm thick sheets. Tests were performed at constant frequency (1 Hz), at controlled strain (0.1%), and with a temperature ramp from −80 to 160 °C at 3 °C/min rate. The preload was set at 0.01 N.

Rheology tests were performed by using an ARES rheometer (TA Instruments, USA) on hot-pressed round specimens of 26 mm of diameter and 1 mm thick. Frequency sweep measurements were performed from 100 to 0.1 rad/s with fixed temperature (150 °C) and strain (1%) after ensuring to work in a viscoelastic field by strain sweep tests. Stability measurements were performed for 1 h at fixed temperature (150 °C), strain (1%), and frequency (1 rad/s). Temperature ramp measurements were performed by using a ramp from 100 to 200 °C at 3 °C/min, with fixed strain (1%) and frequency (1 rad/s).

Tensile tests were performed by using a 5966 dynamometer (Instron, USA). The instrument was equipped with a load cell of 50 N, and the pneumatic clamps were fitted with 25 × 25 mm^2^ flat faces. The materials were prepared by hot-pressing films with a thickness of 250 μm thick and then cut in specimens 20 mm wide and about 100 mm long. The initial distance between the clamps was set at 50 mm and the preload at 0.02 N. The tests were performed at 1 mm/min up to a strain of 0.3% and then at 10 mm/min until specimens broke. Young’s modulus (*E*) was calculated between 0 and 0.1% of strain. The ultimate tensile strength, termed tensile strength for brevity, is σ_max_, and the elongation at break is ε_b_. Three specimens were tested for each material, and the results were averaged.

The evolution of mechanical properties upon multiple recycling steps was studied reprocessing one, two, and three times EPRgF-5.4%BM films by compression molding (15 s at 150 °C) and then annealing them in an oven (3 days at 50 °C) to obtain specimens for tensile testing, according to the method described above.

Thermal conductivity tests were performed through the transient plate source (TPS) method with a 2500S TPS (Hot Disk AB, Sweden), equipped with a Kapton sensor with a radius of 3.189 mm. Measurements were performed by using a bulk method and an impulse of 50 mW for 4 s. Three measurements were taken for each specimen, and the mean value was calculated. The tests were performed keeping the samples at a temperature of 23 °C, which was controlled by a silicon oil bath.

The cross-linking degree was calculated after solubilization tests. About 60 mg of each sample was left in a vacuum oven at 75 mbar overnight, then weighed, and soaked overnight in 20 mL of toluene. After that, the remaining solid part was extracted and dried under the hood. Finally, the sample was left again overnight in a vacuum oven at 75 mbar and then weighed to calculate the percentage of the initial weight to determine the insoluble fraction. The error of the measurement was estimated in the range of 3 wt %. Cross-linking reversibility was investigated by solubilization test, soaking 250 mg of sample in 50 mL of boiling toluene (ca. 110 °C) for 3 h. Nanocomposite samples were subtracted by the rGO contribution to weight, considering only the rubber matrix part. The calculations of cross-linking degree for rubbers without and with rGO are reported in eqs 1 and 2, respectively.
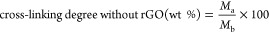
1
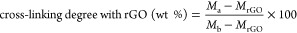
2where *M*_b_ and *M*_a_ are the mass before and after overnight soaking and drying, respectively, and *M*_rGO_ is the mass of the amount of rGO contained in the sample.

## 3 Results and Discussion

### 3.1 Furan Functionalization of EPRgMA via Melt Blending

**Figure 1:**
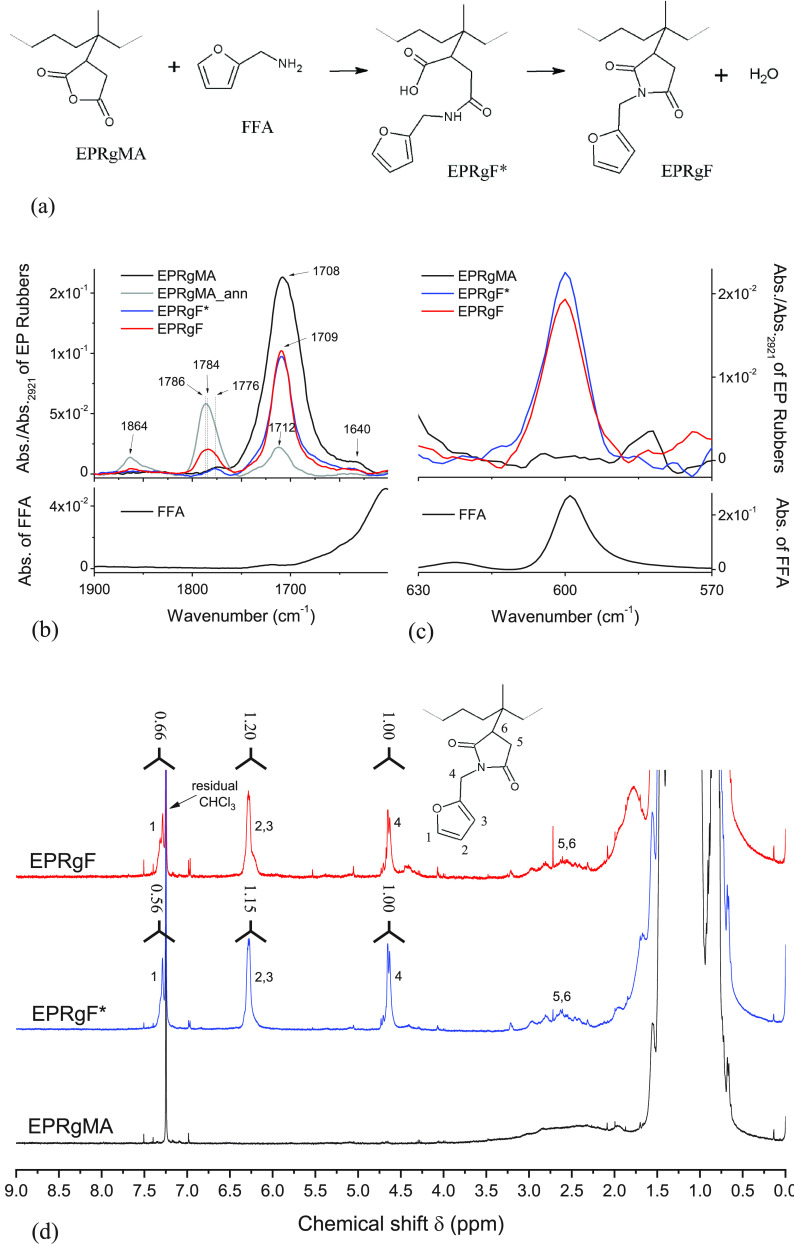
Scheme of the furan functionalization of EPRgMA (a). FT-IR spectra relating to EPRgMA functionalization with FFA in the ranges 1900–1600 cm^–1^ (b) and 630–570 cm^–1^ (c). (Spectra of EP rubbers are normalized on their higher peak at 2921 cm^–1^, assigned to C–H stretching vibration on the aliphatic chain of the rubbers, while FFA spectra are not normalized.) ^1^H NMR (300 MHz, CDCl_3_, room temperature) spectra of EPRgMA, EPRgF*, and EPRgF in the range 4–8 ppm (d).

Figure 1a describes the grafting reaction scheme, starting from the reaction of EPRgMA with FFA, through an amide intermediate and eventually to the furfuryl imide derivative (EPRgF).

Furan grafting on EPRgMA was assessed by FT-IR and ^1^H NMR spectroscopies. With regard to FT-IR spectroscopy, the 1900–1600 cm^–1^ region was primarily taken into account, as the distinctive C=O stretching wavenumbers allow the identification of anhydride groups and its derivatives.

In Figure 1b,c FT-IR spectra of FFA, EPRgMA, EPRgMA_ann, EPRgF*, and EPRgF are reported, while main signal assignment is summarized in Table S1.

In the 1900–1600 cm^–1^ range (Figure 1b), the as-received EPRgMA spectrum shows a strong absorption band centered at 1708 cm^–1^, assigned to C=O stretching of carboxylic acid,^43,44^ confirming the hydrolysis of anhydride groups to dicarboxylic acids.^42^ The EPRgMA_ann spectrum exhibits a decreased carboxylic acid C=O signal at 1712 cm^–1^ and two new bands, at 1864 and 1786 cm^–1^, that are typical of cyclic anhydride C=O asymmetric and symmetric stretching, respectively,^43,44^ supporting the actual conversion to anhydride, via condensation of carboxylic groups under high temperature and reduced pressure.^18^ The IR spectrum for melt mixed EPRgMA was found equivalent to the spectrum for EPRgMA_ann (Figure S1), confirming efficiency of conversion during melt processing. EPRgF* exhibits a strong peak at 1709 cm^–1^, with an intensity intermediate between that of EPRgMA and EPRgMA_ann, which can be explained by residual carboxylic acid groups as well as by the formation of imide groups.^45^ Instead, EPRgF presents a band at 1864 cm^–1^ together with another at 1784 cm^–1^, while a slightly sharper and similarly intense peak at 1709 cm^–1^ was retained compared to EPRgF*. This suggests that the thermal annealing in a vacuum promoted the condensation of carboxyl groups to anhydride and may complete the condensation of possible amide groups with an adjacent carboxyl group to increase the concentration of imide groups. This is further supported by the shape of the broad band centered at 1784 cm^–1^, which is downshifted and broader than for EPRgMA_ann, suggesting an overlap between the signals of C=O symmetric stretching of the anhydride and C=O symmetric stretching of the imide at 1776 cm^–1^.

Overall, these results demonstrate that the grafting of FFA was indeed obtained, although the conversion of available MA groups was not quantitative, despite excess of amine, which likely depends on the competition between the amine–anhydride reaction and the FFA evaporation under melt blending conditions. Further evidence for the achievement of furan grafting on the rubber chain can be obtained by the careful analysis of the FT-IR region for furan ring deformation. Indeed, the band at 599 cm^–1^ in the FFA spectrum (Figure 1c) was previously assigned to furan ring deformation.^43,46^ The same signal is clearly visible in EPRgF* and EPRgF spectra, thus confirming the furan grafting on the EPR polymer chains.

To provide further evidence for furan functionalization on the rubber chain, ^1^H NMR spectroscopy measurements were performed, focusing on the signals of furan protons. In Figure 1d, the ^1^H NMR spectra of EPRgMA, EPRgF*, and EPRgF in the range 4–8 ppm are reported. Conversely, for the neat EPRgMA, new signals appear at 7.29, 6.28, and 4.64 ppm in the spectra of the two treated samples (ERPgF* and EPRgF). The first two signals can be attributed to protons of the furan ring,^47^ while the third can be due to methylene protons of the imide.^16^ These results confirm the grafting of the furan group, proving that the formation of the imide occurs, at least partially, before the annealing treatment.

### 3.2 Thermoreversible Cross-Linking of EPRgF with BM

**Figure 2:**
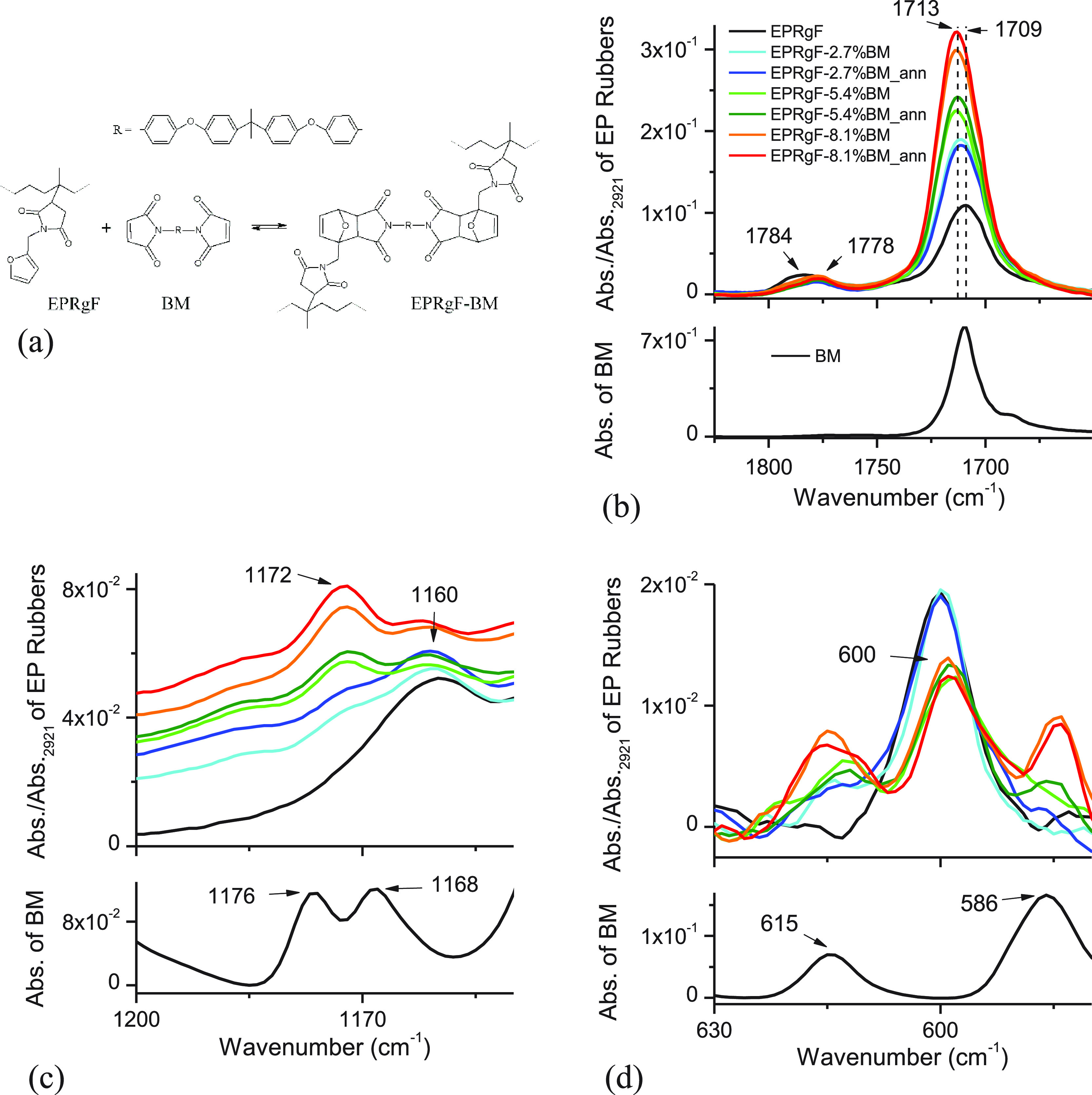
Scheme of EPRgF cross-linking with BM (a). FT-IR spectra of EPRgF and its cross-linked networks in the ranges 1825–1650 cm^–1^ (b), 1200–1150 cm^–1^ (c), and 630–580 cm^–1^ (d).

To obtain a thermoreversible cross-linking of the rubber, BM was blended with EPRgF in different percentages. In Figure 2a, the scheme of the cross-linking reaction is reported.

The morphology of EPRgF-BM was routinely observed by SEM imaging (Figure S2), which did not show evidence for phase separation at the microscale, regardless of the concentration of BM, thus confirming a homogeneous distribution of BM.

DSC was also performed as an attempt to identify temperatures for DA and retro-DA reactions:^48^ however, the thermograms for the different EPRgF-BM (Figure S3) do not show clear signals for DA transitions, likely due to the low concentration of cross-linking and the limited sensitivity of the DSC technique.

To detect DA adduct formation between furan and maleimide moieties, FT-IR spectra of the EPRgF-BM compounds were studied in details (Figure 2b–d).

In the C=O stretching region (Figure 2b), the BM spectrum shows a strong band at 1710 cm^–1^, assigned to the C=O asymmetric stretching of maleimide. EPRgF-BM spectra exhibit a strong C=O asymmetric stretching signal centered around 1713 cm^–1^, which appears related to the presence of the imide in BM, as these signals increase in intensity with increasing the BM concentrations and are not significantly modified by thermal annealing. Moreover, all the EPRgF-BM spectra show a C=O symmetric stretching at 1778 cm^–1^, which is significantly shifted compared to the EPRgF (1784 cm^–1^) spectrum. This behavior may be related to the formation of the DA adduct between furan and maleimide, which generates a change of the C=O vibration mode of cyclic imides, depending on their chemical environment, in agreement with a previous literature report.^49^ FT-IR spectra in the range 1200–1150 cm^–1^ (Figure 2c) were also studied, as the C–O–C stretching band in furan ring can be observed at 1160 cm^–1^ in EPRgF.^43^ In the EPRgF-BM spectra, such a signal appears of lower intensity, while a peak at 1172 cm^–1^ is clearly visible of increasing intensity with BM concentration and almost independent of the thermal annealing. This band does not seem correlated to the bands in BM spectrum at 1176 and 1168 cm^–1^, and it was previously associated with a shift of C–O–C stretching due to DA adduct formation.^49^ In the low FT-IR wavenumber region (Figure 2d), the BM spectrum shows two peaks at 615 and 586 cm^–1^, which do not overlap significantly with furan ring deformations in the region of 600 cm^–1^. In the EPRgF-BM spectra, the characteristic peak of BM at ∼586 cm^–1^ is clearly visible, increasing in intensity with BM concentration. On the other hand, a decrease of the peak at 600 cm^–1^, assigned to furan ring deformation, was found, which further supports the formation of the DA adduct.

With the aim of proving accomplished cross-linking in the rubber, solubility tests were carried out, knowing both EPRgMA and EPRgF are highly soluble in toluene at room temperature. Conversely, all the BM-added rubbers turned out to be poorly soluble in the same solvent. Instead, the EPRgF-BM showed swelling in toluene, and the solid residue was collected to determine the cross-linking degree (Figure S4) for each formulation. The cross-linking degree increases up to about 85% with 8.1 wt % BM content. Furthermore, the thermal annealing brings an increase of cross-linking degree for the lower BM concentrations, evidencing for a slight optimization of cross-linking when the materials is given time to complete DA adduct formation, whereas variation within the experimental error was found for EPRgF-8.1%BM. These results suggest maximum cross-linking is reached at 8.1% BM, which cannot be enhanced further by thermal annealing.

**Figure 3:**
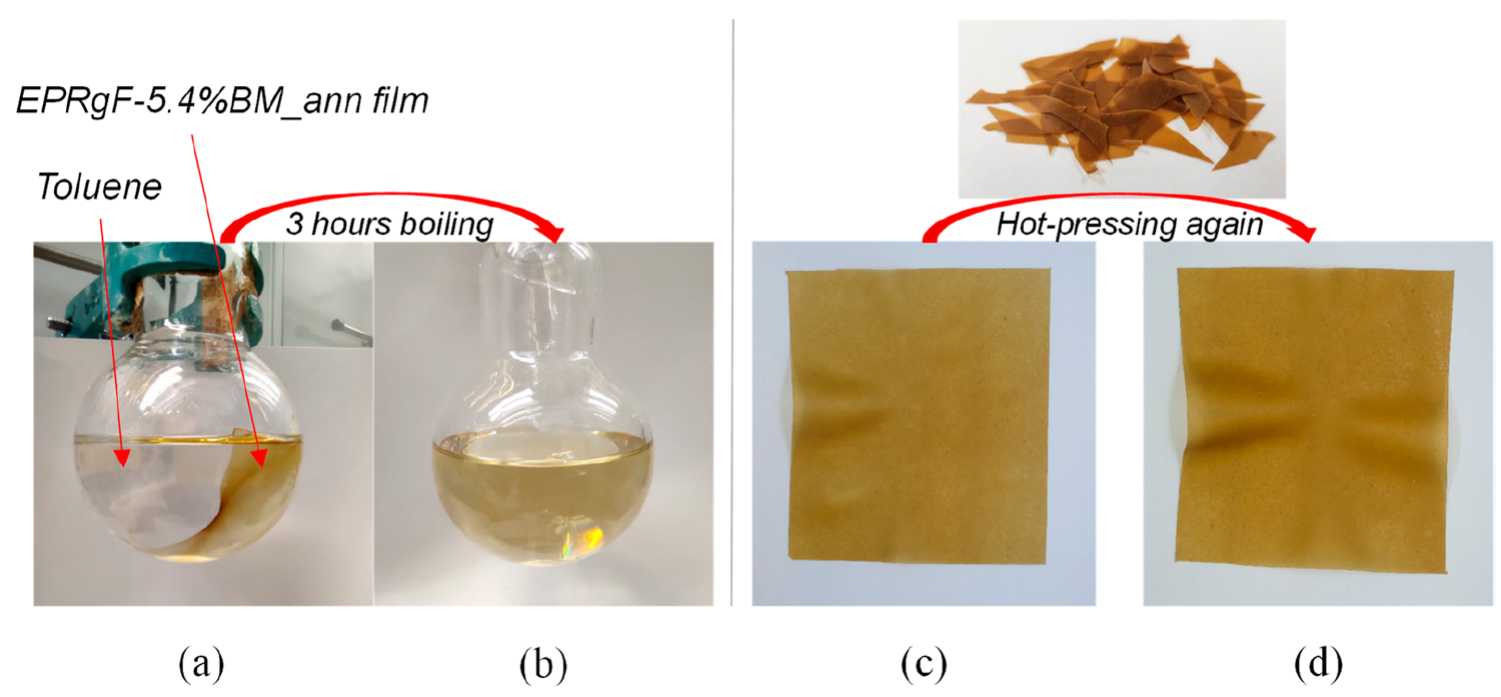
EPRgF-5.4%BM_ann film in toluene before and after 3 h boiling. A solid film is clearly visible in (a) whereas a solution in (b). Remolding via hot-pressing of a EPRgF-5.4%BM film (c) and a new one (d).

To prove the reversible character of DA cross-linking, the solubility test on EPRgF-5.4%BM_ann in boiling toluene has also been performed. It resulted that the cross-linked formulation, with an insoluble fraction at room temperature, after 3 h in boiling toluene was completely solubilized. In Figure 3, pictures of a EPRgF-5.4%BM_ann film soaked toluene were captured before (a) and after (b) 3 h in boiling toluene.

The great difference in solubility at room temperature vs high temperature (ca. 110 °C) evidences for the achievement of a covalent cross-linking at room temperature, yet thermoreversible via retro-DA reaction at high temperature. To bring further evidence of reversible cross-linking, EPRgF-BM films were chopped and hot-pressed again at 150 °C into a new film. It turned out that it was always possible to obtain homogeneous films (Figure 3c,d), suggesting the material flows similarly to a thermoplastic polymer, thanks to the promotion of a retro-Diels–Alder reaction at this temperature and to the consequent temporary breaking of cross-links.

To further investigate the rubber behavior at processing temperature (150 °C), rheology tests were performed at the same temperature.

**Figure 4:**
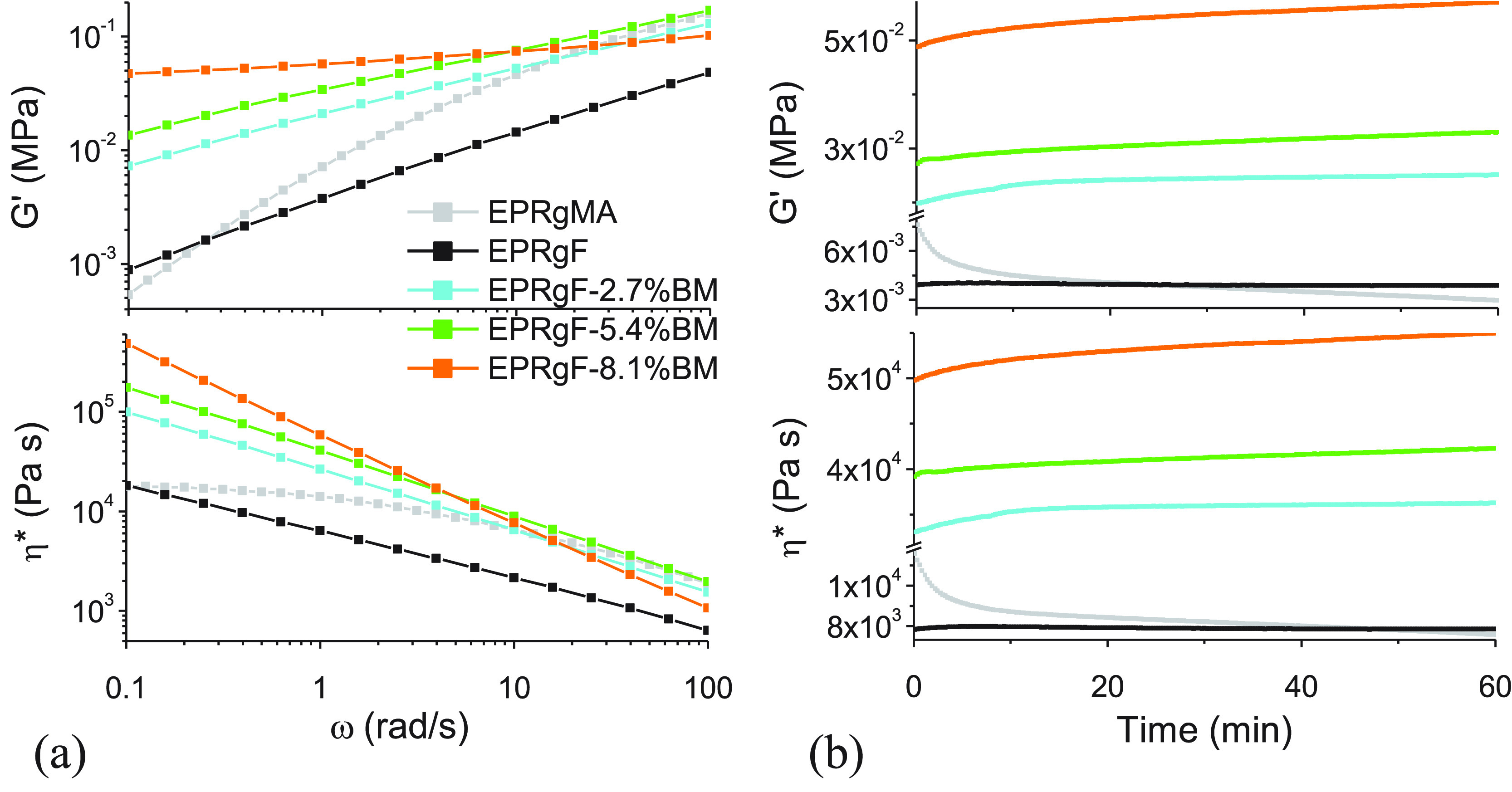
Storage modulus (*G*′) and complex viscosity (η*) in function of angular frequency (ω) (a) and time (b), at constant temperature and strain, for EPRgMA, EPRgF, and EPRgF-BM networks.

In the frequency sweep mode (Figure 4a), *G*′ plots for EPRgF-BM networks show a significantly lower slope at low frequencies compared to EPRgMA and EPRgF, which is consistent to a cross-linking character, bringing evidence of the presence of DA adducts linking the chains even at 150 °C. This is further confirmed by the lower slope for EPRgF-8.1%BM compared to EPRgF-2.7%BM and EPRgF-5.4%BM, which is in agreement with the higher cross-linking degree observed at low temperature. Complex viscosity curves for the rubber networks show high viscosity at low frequencies, ∼1 order of magnitude higher than EPRgMA and EPRgF, with a progressive increase with the content of BM (consistent with *G*′ curves) and clear shear thinning behavior leading to a drastic decrease of viscosity with increasing frequency. Isothermal (150 °C) stability measurements (Figure 4b) confirm these observations, showing a progressive annealing of the samples, with slightly increasing *G*′ and η* with time. Indeed, while the retro-DA was shown to be thermally activated at lower temperature (Figure 3), it should be noted that the DA is an equilibrium reaction, which can be shifted to the dissociated side when increasing temperature,^50^ while still retaining a fraction of bound chains. These contribute to the high viscosity and solidlike behavior of the EPRgF-BM formulations, especially when containing relatively high BM concentrations. While partial cleavage of the network may be sufficient to solubilize the rubber in a solvent at relatively low temperature, reassociation of furan and maleimide moieties is strongly favored in the molten state, which therefore requires significantly higher temperatures to obtain a melt-processable fluid, also taking into account the viscosity of the pristine polymer. In fact, despite DA-cross-linked polymers being traditionally considered as dissociative networks, these present some similarities with an associative dynamic network, where the cross-link density depends on the temperature and in principle remains constant during processing. Therefore, in this case, boundaries between covalent associative and covalent dissociative networks do not appear well-defined, which was in fact recently discussed already for 1,2,3-triazolium-based dissociative networks.^51^

**Figure 5:**
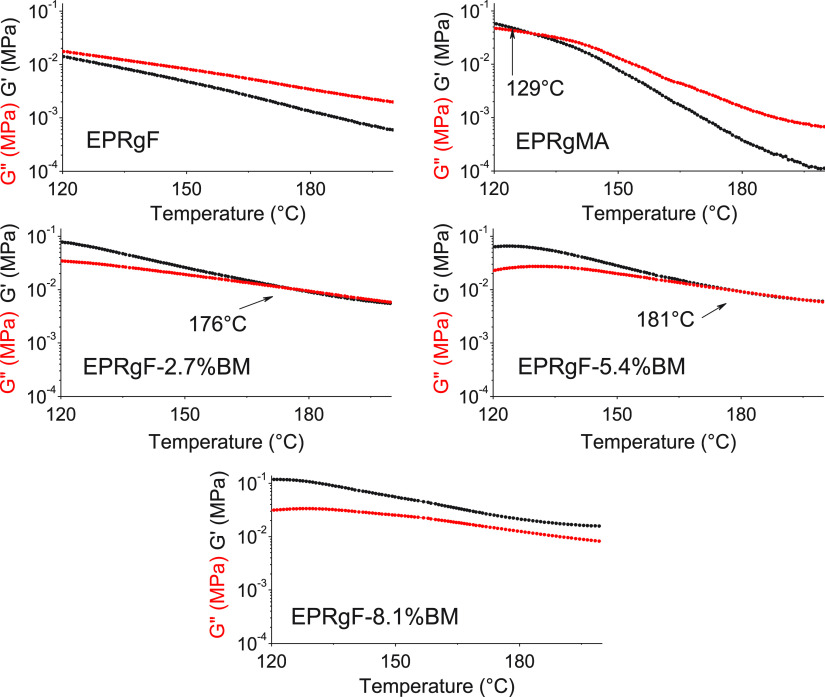
Storage modulus (*G*′) and loss modulus (*G*″) as a function of temperature, at constant frequency and strain, for EPRgMA, EPRgF, and EPRgF-BM networks.

To further investigate dynamic cross-linking in EPRgF-BM rubbers as a function of temperature, rheology measurements in the temperature ramp were performed (Figure 5).

EPRgF shows a liquidlike behavior as expected as these temperatures, with *G*″ > *G*′ over the whole temperature range. The higher *G*′ of EPRgMA at low temperatures may be explained by hydrogen bonding between chains through carboxylic and/or anhydride groups, which are not possible or strongly limited in EPRgF, where the majority of MA groups were previously reacted with FFA. In EPRgF-BM, *G*′ is dominant over the whole temperature range at 8.1% BM content, whereas a transition from solidlike to liquidlike behavior is found at lower BM contents. The temperature at which this transition is observed depends on the BM content, namely 176 °C for EPRgF-2.7%BM and 181 °C for EPRgF-5.4%BM), which is significantly higher than the temperature (ca. 110 °C) sufficient to dissolve the rubber in solvent. This confirms an equilibrium state for the DA reaction in the melt, which is shifted in temperature and depends on the concentration of BM, namely increasing the effective cross-linking at 150 °C with increasing BM content.

**Figure 6:**
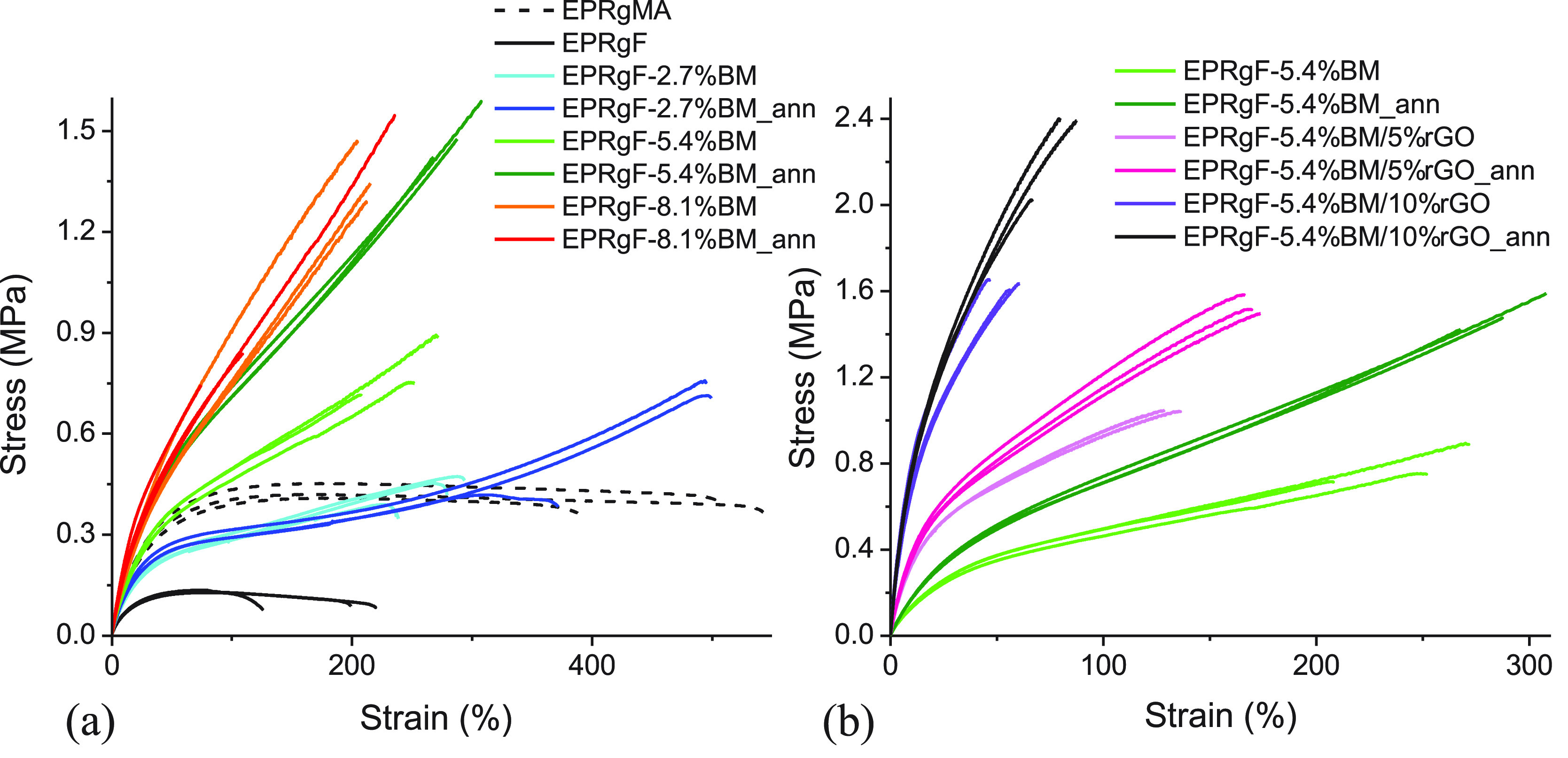
Stress–strain curves of EPRgMA, EPRgF, and EPRgF-BM networks (a) and rGO nanocomposites (b).

At room temperature, the effect of cross-linking on the rubber has been also investigated through its mechanical properties, performing tensile testing on EPRgMA, EPRgF, and EPRgF-BM with different amounts of cross-linking agent, both before and after thermal annealing. Figure 6a reports the resulting stress–strain curves of the three EPRgF-BM formulations, both as obtained and after a thermal annealing. In Table 2, the corresponding average Young’s modulus (*E*), tensile strength (σ_max_), and elongation at break (ε_b_) are reported with their standard deviations.

**Table 2:** Average Young’s Modulus *E*, Tensile Strength σ_max_, and Elongation at Break ε_b_ with Their Standard Deviations of EPRgMA, EPRgF, EPRgF-BM, and EPRgF-BM/rGO

	av ± st dev of *E* (MPa)	av ± st dev of σ_max_ (MPa)	av ± st dev of ε_b_ (%)
EPRgMA	1.14 ± 0.46	0.43 ± 0.02	482 ± 78
EPRgF	0.49 ± 0.14	0.13 ± 0.00	182 ± 49
EPRgF-2.7%BM	1.05 ± 0.14	0.44 ± 0.04	270 ± 29
EPRgF-2.7%BM_ann	1.11 ± 0.10	0.63 ± 0.18	455 ± 73
EPRgF-5.4%BM	1.38 ± 0.10	0.79 ± 0.09	244 ± 33
EPRgF-5.4%BM_ann	1.44 ± 0.26	1.50 ± 0.09	288 ± 20
EPRgF-8.1%BM	1.37 ± 0.28	1.36 ± 0.09	211 ± 5
EPRgF-8.1%BM_ann	1.68 ± 0.11	1.04 ± 0.44	140 ± 85
EPRgF-5.4%BM/5%rGO	3.91 ± 0.30	1.04 ± 0.02	129 ± 8
EPRgF-5.4%BM/5%rG _ann	4.16 ± 0.08	1.53 ± 0.04	170 ± 4
EPRgF-5.4%BM/10%rGO	8.30 ± 1.07	1.64 ± 0.03	55 ± 7
EPRgF-5.4%BM/10%rGO_ann	8.14 ± 1.78	2.27 ± 0.21	78 ± 10

EPRgMA is a soft and highly stretchable polymer, displaying an *E* of ∼1.14 MPa and elongation at break on the order of 500%. A drastic decrease of Young’s modulus, elongation, and tensile strength was observed for EPRgF compared to EPRgMA. The higher performances of EPRgMA are explained accordingly with rheology measurements at low temperatures by hydrogen bonding between chains. With regard to BM-cross-linked polymers, a progressive reinforcement of the rubber was obtained with the concentration of BM, with clear enhancement of the elastic modulus (∼1.44 MPa for EPRgF-5.4%BM_ann) and tensile strength (∼1.50 MPa for the same material vs 0.43 MPa for EPRgMA), along with a decrease in elongation at break. Upon annealing, a general enhancement in mechanical properties was obtained, with differences depending on the BM concentration. At low BM content (2.7 wt %), annealing does not lead to significant improvements in modulus and thickness, but elongation at break increases significantly, possibly related to recovery of microdefects during annealing. On the other hand, EPRgF-5.4%BM exhibits a significant enhancement in tensile strength upon annealing (approximately from 0.79 to 1.50 MPa), which appears to confirm the increase in the cross-linking density after the thermal treatment. EPRgF-8.1%BM presents similar properties compared to annealed EPRgF-5.4%BM, but its annealing leads to a limited enhancement in the modulus and a reduction in elongation. This is consistent with the unmodified cross-linking degree for EPRgF-8.1%BM after annealing (Figure S4).

To investigate the evolution of mechanical properties upon material recycling, multiple reprocessed EPRgF-5.4%BM was tested again in tensile mode. The results obtained, reported in Figure S5 and Table S2, evidence a progressive increase in stiffness, leading to a 1.92 ± 0.13 MPa modulus after three recycling steps compared to 1.44 ± 0.26 MPa for pristine EPRgF-5.4%. The tensile strength was also slightly increased from 1.50 ± 0.09 MPa to 1.86 ± 0.15 MPa after the three recycling steps, whereas the elongation at break remain almost constant in the range of 300%.

With the aim of exploring viscoelastic properties of the rubber DMTA measures were performed, as reported in Figure S6. The glass transition temperature of the rubbers (pristine, furan-functionalized, and cross-linked) has been assessed around −47 °C, with the only exception of EPRgF-8.1%BM_ann, which presents a slight increase (−43 °C). The materials do not show other transitions in the analyzed temperature range, bringing evidence of their monophasic nature, consistent with SEM analysis. Starting at −20 °C, the materials present a clear rubber *plateau* for both storage and loss moduli. It is worth noting that EPRgF shows a decrease of the storage modulus *plateau* with respect to EPRgMA (−65% at 20 °C), while adding BM results in its progressive increase (+225% at 20 °C for EPRgF-8.1%BM_ann, with respect to EPRgF, confirming tensile tests results) and its slower decay at higher temperatures.

### 3.3 EPRgF-BM-rGO Nanocomposites

**Figure 7:**
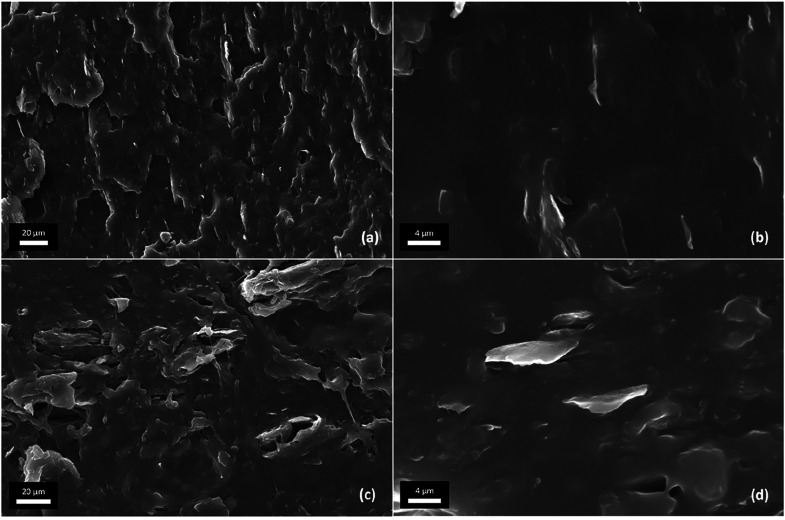
SEM images of EPRgF-5.4%BM/5%rGO (a, b) and EPRgF-5.4%BM/10%rGO (c, d).

To enhance stiffness, tensile strength, and thermal conductivity of the cross-linked rubber, rGO was added to prepare thermoreversible cross-linked nanocomposites. In Figure 7 SEM micrographs of the prepared nanocomposites are reported.

SEM micrographs show evidence of good distributions and dispersions of rGO at the microscale in 5 wt % composite (Figure 7a), whereas more aggregates appear in the composite with 10 wt % rGO (Figure 7c), probably due to the shorter mixing time allowed before blocking of the microextruder. In both the composites at higher magnification (Figure 7b,d), rGO *lamellae* sizing on the order of micrometers are clearly visible and typically oriented in the direction of flow during mixing/extrusion.

To check the cross-linking degree of the nanocomposites, these were subjected to a solubilization test (Figure S4). EPRgF-5.4%BM/5%rGO shows a cross-linking degree similar to EPRgF-5.4%BM, without any further contribution of annealing. Adding rGO brings a slight but noteworthy enhancement of cross-linking degree (from 75% to 81%) in EPRgF-5.4%BM/10%rGO, suggesting an active role of rGO in the covalent cross-linking of the material through DA reaction with BM.

**Figure 8:**
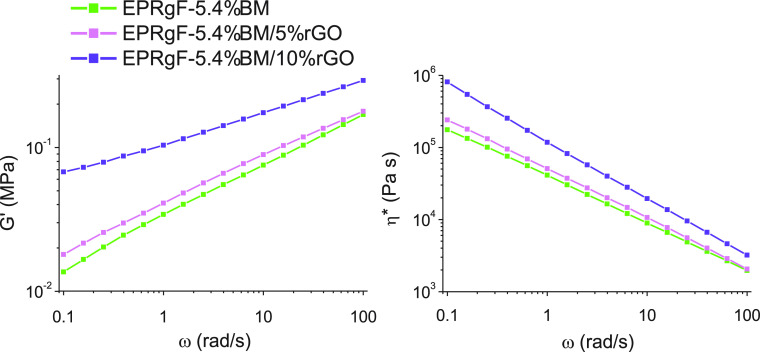
Storage modulus (*G*′) and complex viscosity (η*) as a function of angular frequency (ω) for EPRgF-5.4%BM and its nanocomposites.

To further investigate rGO dispersion in the composites, rheology was addressed (Figure 8). Indeed, rheology provides information about the organization of nanoflakes in the bulk, complementing local analyses obtained by SEM.^52^

The addition of 5%rGO to EPRgF-5.4%BM brings limited increase to *G*′ and η* plots, whereas much higher effects are observed when adding 10%rGO. Indeed, EPRgF-5.4%BM/10%rGO exhibits a lower slope of *G*′ at low angular frequencies, which may be explained by the physical percolation of rGO, in addition to the interactions between BM-bound EPRgF chains. The presence of an effect of physical percolation is an indicator of a good distribution of rGO in the matrix,^52^ as observed by SEM in EPRgF-5.4%BM/5%rGO (Figure 7a) and bringing evidence of better dispersion than the SEM-observed one for EPRgF-5.4%BM/10%rGO (Figure 7c).

Mechanical properties of the nanocomposites were investigated by a tensile test in the same conditions as the corresponding EPRgF-BM. The stress–strain curves are shown in Figure 6b, and the corresponding average Young’s modulus (*E*), tensile strength (σ_max_), and elongation at break (ε_b_) are reported in Table 2, with their standard deviations. In nanocomposites the inclusion of rGO determines a remarkable increase in stiffness (+189% at 5%rGO and +465% at 10%rGO) and tensile strength (σ_max_ + 51% at 10%rGO) compared to the annealed samples of nanocomposites and reference matrix, at the expense of a strong decrease in elongation at break (i.e., about one-fourth at 10%rGO). The effect of annealing is similar to that observed in the matrix, bringing an enhancement in tensile strength (+38% for EPRgF-5.4%BM/10%rGO) and elongation at break without notable effects on stiffness. Only a few papers reported mechanical properties of nanocomposites in DA cross-linked matrices. Jia et al.^31^ reported cross-linking of EPRgF on dienophile-functionalized silica, leading to a 2.5 MPa tensile strength and 800% fracture strain, when adding 13 wt % functionalized silica. Polgar et al. reported BM-cross-linked EPRgF filled with carbon nanotubes (CNT),^34^ yielding significantly higher tensile strength (7 MPa) with 10 wt % of CNT, together with an 80% elongation at break. Results obtained in this work using rGO are comparable to the previous reports: for instance, EPRgF-5.4%BM/10%rGO_ann has a 2.3 ± 0.2 MPa tensile strength with ∼80% fracture strain, which appears competitive with previously reported nanocomposites, especially when considering the materials in this work were obtained by melt processing instead of the conventional solvent mixing.

To investigate the thermal conductivity of the composites, TPS measures were performed on the different formulations. The thermal conductivity of pristine EPRgMA (0.21 ± 0.01 W/(m K)) is almost unmodified by the furan grafting and cross-linking, yielding 0.24 ± 0.01 for EPRgF and EPrgF-5.4%BM. On the other hand, addition of rGO gives to the rubber a clear enhancement of thermal conductivity, which rises to 0.35 ± 0.02 W/(m K) (+70%) with 5 wt % rGO and 0.47 ± 0.05 W/(m K) (+124%) with 10 wt % rGO compared to the pristine rubber, with no significant effect of thermal annealing. The thermal conductivity results are summarized in Figure S7. The thermal conductivity obtained in this work is higher than reported in rubbers filled with other conductive particles: in particular, hexagonal boron nitride (14 wt %, particles of 2–3 μm) in siloxane rubber yielded 0.4 W/(m K),^53^ whereas expanded graphite (9 wt %, particles ca. 300 μm) in silicone rubber exhibited a 0.25 W/(m K) conductivity.^54^ Therefore, inclusion of rGO appears as a promising strategy to enhance rubber thermal conductivity, keeping the filling percentage low. This is in fact required in application where high flexibility and deformability to high strain are required, such as in modern flexible electronics,^55^ including next generations of wearable and implantable devices.^56,57^

## 4 Conclusions

In this work, we prepared a thermally reversible cross-linked ethylene–propylene rubber (EPR), for the first time through melt processing, as a sustainable alternative to the preparation in organic solvents. Indeed, a maleated EPR was grafted via melt blending to introduce furan groups and then reacted in a second melt blending step with bismaleimide (BM) moieties to produce covalently cross-linked polymers via the Diels–Alder (DA) reaction. Cross-linking degrees were evaluated by solvent extraction and found directly dependent on the BM concentration, up to ∼85% with 8.1 wt % BM. The cross-linking degree is expectedly reflected in the mechanical properties at room temperature, exhibiting higher stiffness and tensile strength with increasing BM content, still yielding soft and highly deformable rubbers, with elastic moduli in the range of 1 MPa and elongation at break typically higher than 200%. Thanks to the thermally cleavable bonds via the retro-Diels–Alder reaction, cross-linked materials were demonstrated melt-reprocessable into new films. The thermally activated cleavage of the network was first proven by solubilization test at 110 °C, whereas reprocessing as a melt required higher temperatures, in the range of 150 °C. This is because of both the pristine polymer viscosity and the nature of the dissociative reaction. Indeed, because the retro-DA reaction defines an equilibrium state, the full dissociation of the network is not possible in the molten state, but the equilibrium can be sufficiently shifted toward the dissociated form to allow melt reprocessing. The full recyclability of DA-cross-linked rubbers was demonstrated via multiple reprocessing via compression molding and tensile testing, showing stiffness and strength are slightly increased upon multiple recycling, while elongation at break is maintained.

The reversible cross-linked rubber was also demonstrated for the possible mechanical reinforcement with carbonaceous nanofillers. In particular, reduced graphene oxide (rGO) was added at 5 or 10 wt % loading, aiming at the preparation of reinforced and thermally conductive elastomers. The results indicated homogeneous dispersion of rGO and a remarkable increase in stiffness (up to 8 MPa) and tensile strength (up to 2 MPa) compared to the case of unfilled rubber, along with a thermal conductivity up to 0.47 W/(m K), while retaining melt reprocessability.

The implementation of thermally reversible cross-linking in rubbers, as demonstrated in this work, brings clear advantages in terms of recyclability of rubbers, opening for the substitution of traditionally cross-linked elastomers. Compared to the previously reported methods, the environmentally friendly gain is increased by the solvent-less process, which represents a cost-effective and industrially viable solution.

## Supporting Information Available

Table S1: FT-IR spectra assignment; Figure S1: FT-IR spectra of EPRgMA subjected to annealing; Figure S2: SEM images of EPRgF-BM networks; Figure S3: DSC thermograms of EPRgMA, EPRgF, and EPRgF-BM networks; Figure S4: cross-linking degree obtained by solubilization tests; Figure S5: stress–strain curves of reprocessed EPRgF-5.4%BM_ann specimens; Table S2: average Young’s modulus, tensile strength, and elongation at break of reprocessed EPRgF-5.4%BM_ann specimens; Figure S6: DMTA curves of EPRgMA, EPRgF, and EPRgF-BM networks; Figure S7: thermal conductivity values (PDF)
