# Supplementary material for: Thermoreversible Cross-Linked Rubber Prepared via Melt Blending and Its Nanocomposites
Source: ACS Appl Polym Mater. 2022 Jun 17;4(7):4796–807. doi: 10.1021/acsapm.2c00416 (PMC9274616; doi:10.1021/acsapm.2c00416)
Supplement: Supplementary file 1 — ap2c00416_si_001.pdf [file ap2c00416_si_001.pdf]

SUPPORTING INFORMATION

Thermo-reversible crosslinked rubber prepared via melt blending and its nanocomposites

*Francesco Cantamessa [a], Giacomo Damonte [b], Orietta Monticelli [b], Rossella Arrigo [a], Alberto Fina [a, *]*

[a] Dipartimento di Scienza Applicata e Tecnologia, Politecnico di Torino, 15121 Alessandria, Italy

[b] Dipartimento di Chimica e Chimica Industriale, Università di Genova, 16146 Genova, Italy

* Corresponding author. E-mail address: alberto.fina@polito.it

Table S1. Assignment of FT-IR spectra for EPRgF and EPRgF-BM characterization.

**Wavenumber** *(cm^{-1})*	**Assignment**
~ 1864	C=O asymmetric stretching of maleic anhydride
1786-1784	C=O symmetric stretching of maleic anhydride
1776-1778	C=O symmetric stretching of imide
1709-1713	C=O asymmetric stretching of imide
1712-1708	C=O stretching of carboxylic acid
1172-1160	C-O-C stretching in furan ring
~ 600	Furan ring deformation

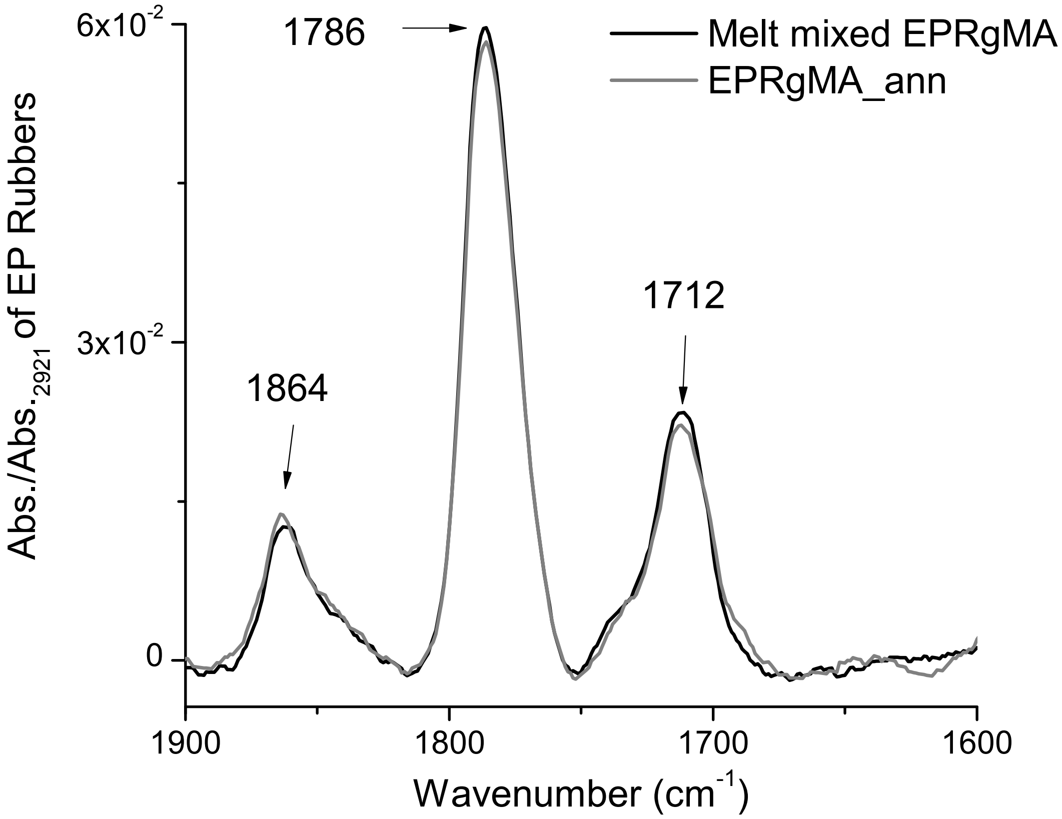

Figure S1. FT-IR spectra in the range 1900-1600 cm^{-1} of EPRgMA after melt processing and after annealing.

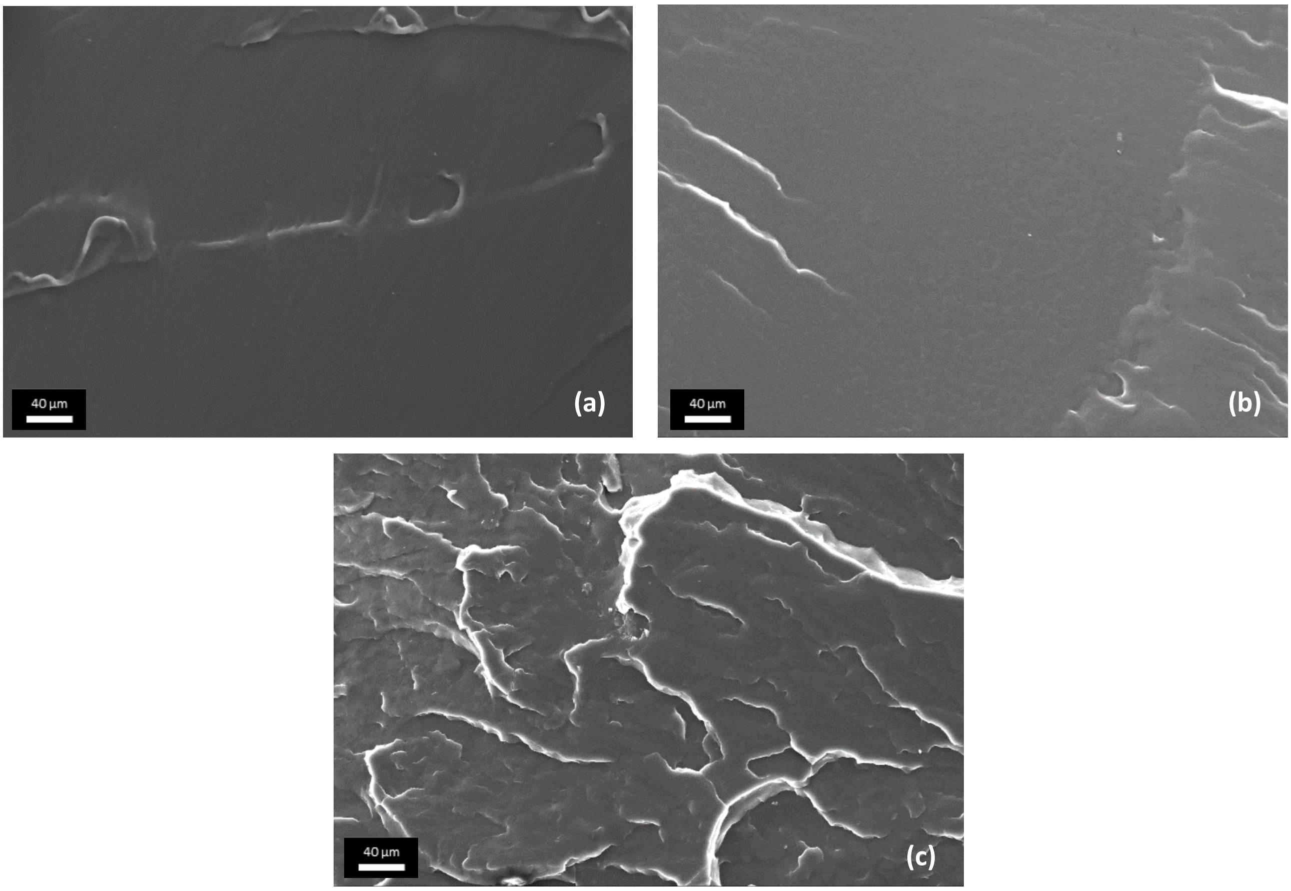

Figure S2. SEM images of EPRgF-2.7%BM (a), EPRgF-5.4%BM (b) and EPRgF-8.1%BM (c).

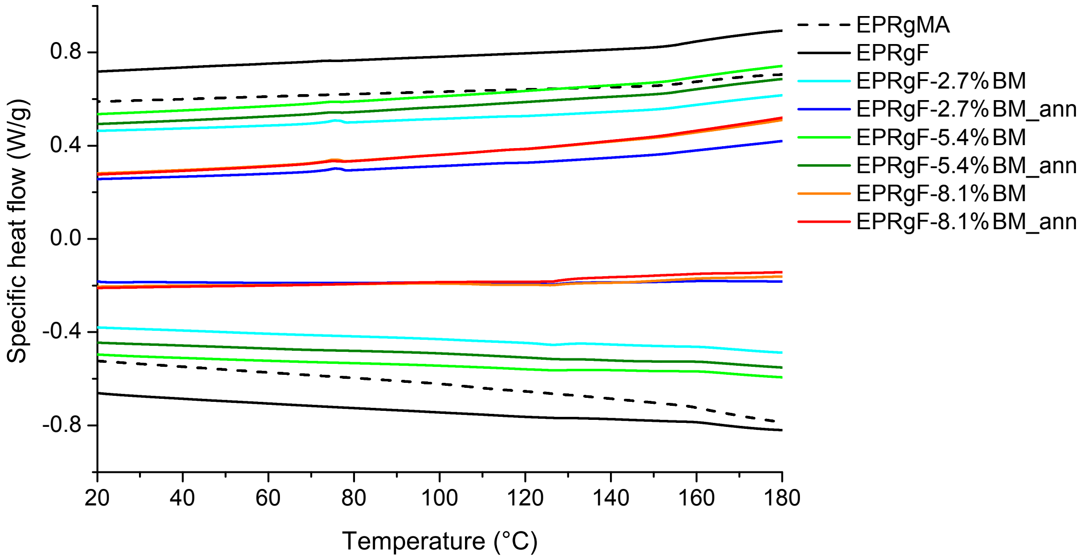

Figure S3. DSC thermograms of EPRgMA, EPRgF and EPRgF-BM.

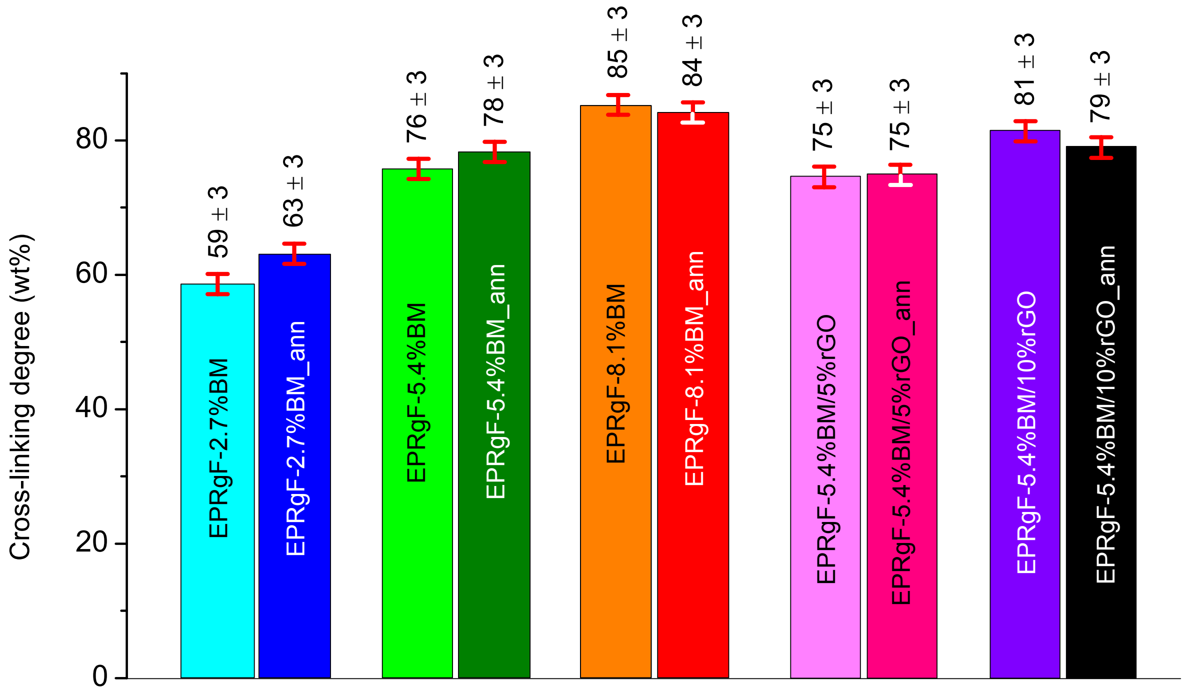

Figure S4. Percentage of remained rubber and nanocomposite after overnight soaking in toluene.

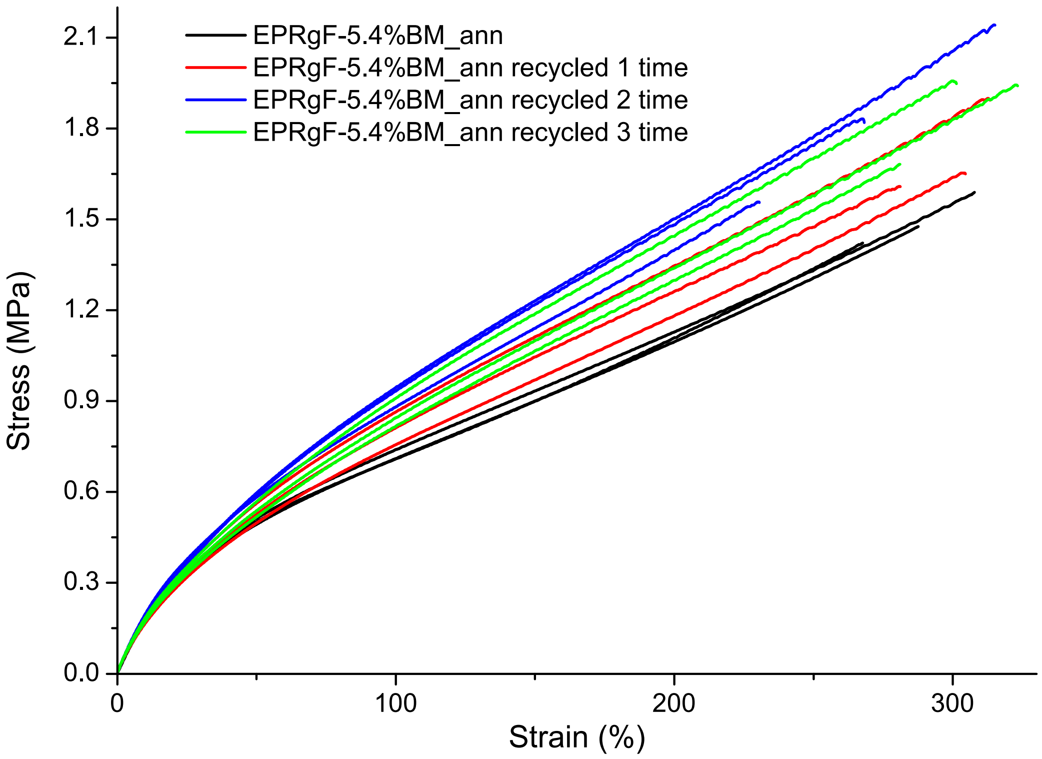

Figure S5. Stress-strain curves of EPRgF-5.4%BM_ann, reprocessed 3 times.

Table S2. Average Young's modulus E, tensile strength σ_{max} and elongation at break ε_b with their standard deviations of EPRgF-5.4%BM_ann, reprocessed 3 times.

	**Average ± *st. dev.* of E** (MPa)	**Average ± *st. dev.* of σ_{max}** (MPa)	**Average ± *st. dev.* of ε_b** (%)
EPRgF-5.4%BM_ann	1.44 ± *0.26*	1.50 ± *0.09*	288 ± *20*
EPRgF-5.4%BM_ann recycled 1 time	1.77 ± *0.16*	1.72 ± *0.16*	300 ± *16*
EPRgF-5.4%BM_ann recycled 2 time	1.84 ± *0.05*	1.84 ± *0.29*	271 ± *42*
EPRgF-5.4%BM_ann recycled 3 time	1.92 ± *0.13*	1.86 ± *0.15*	301 ± *21*

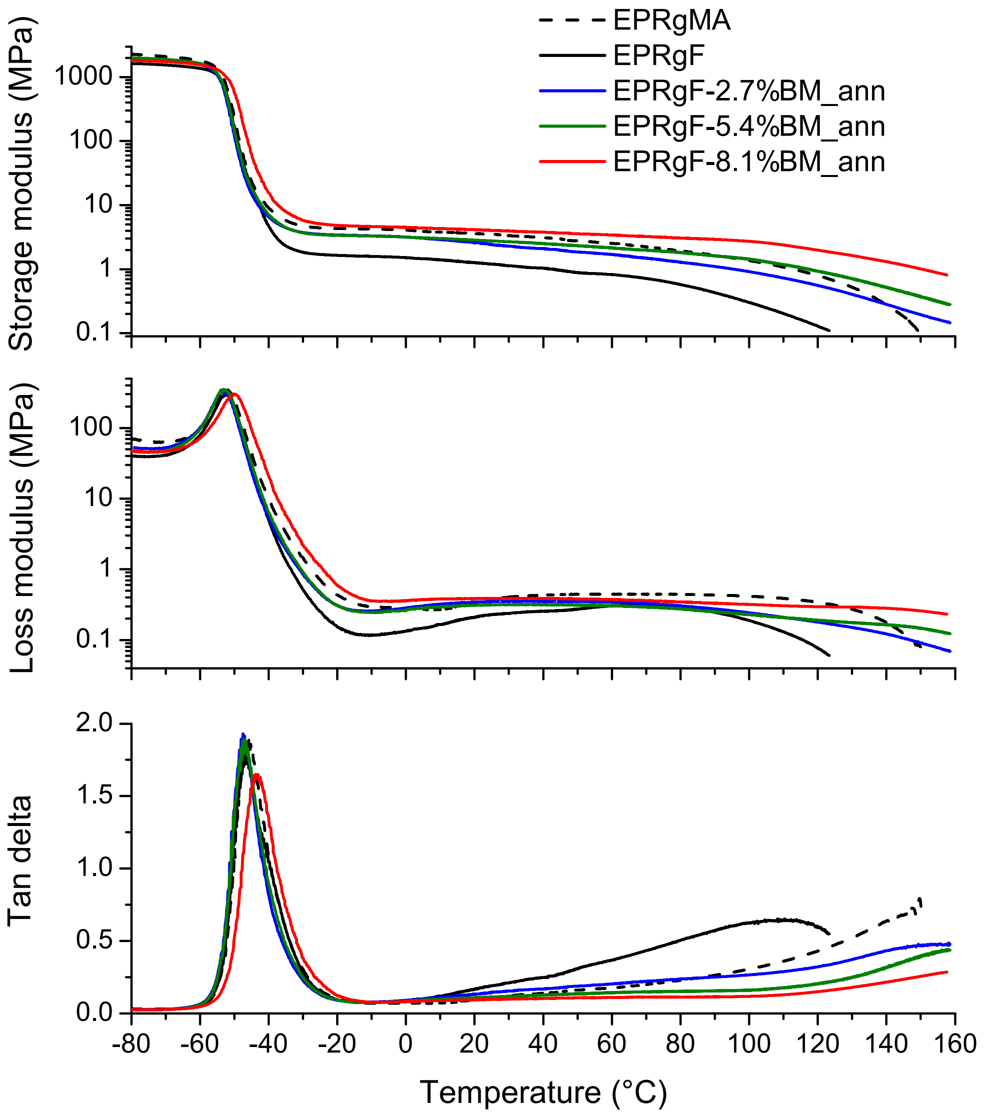

Figure S6. DMTA curves of EPRgMA, EPRgF and its BM crosslinked networks.

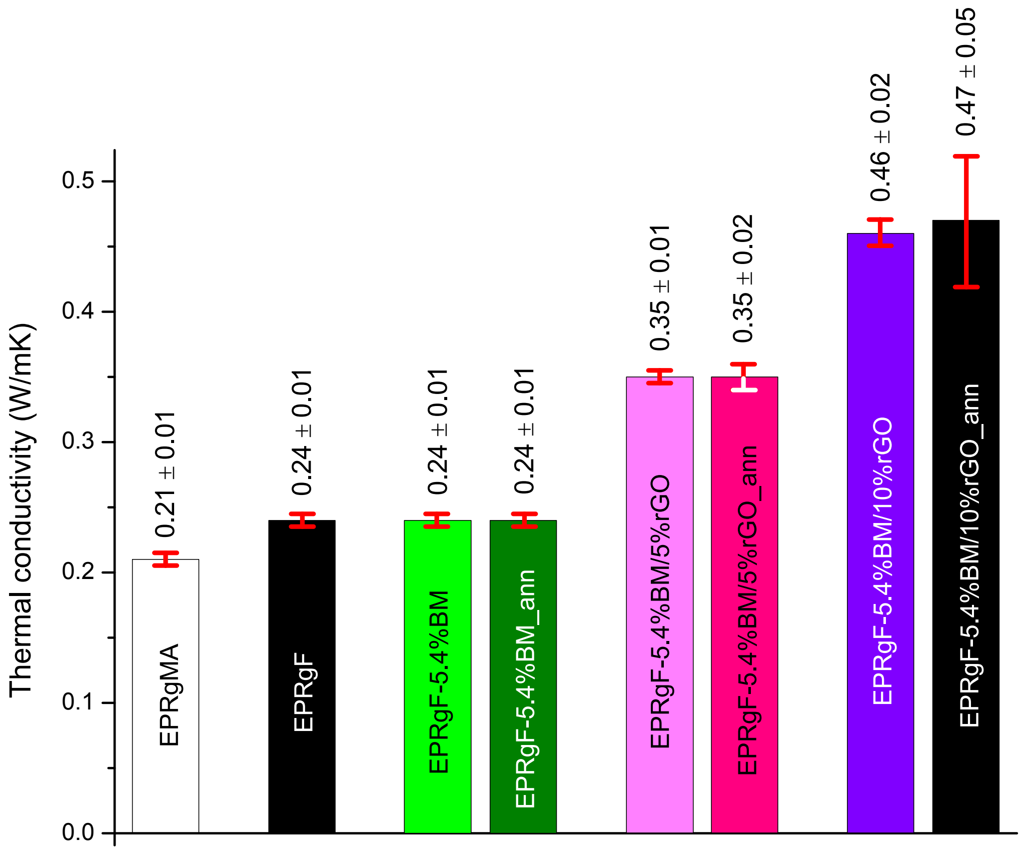

Figure S7. Thermal conductivity values of rubbers and rGO composites with their standard deviation (red bar).
